# Non-thermal Plasma Causes p53-Dependent Apoptosis in Human Colon Carcinoma Cells

**Published:** 2012

**Authors:** A.I. Tuhvatulin, E.V. Sysolyatina, D.V. Scheblyakov, D.Yu. Logunov, M.M. Vasiliev, M.A. Yurova, M.A. Danilova, O.F. Petrov, B.S. Naroditsky, G.E. Morfill, A.I. Grigoriev, V.E. Fortov, A.L. Gintsburg, S.A. Ermolaeva

**Affiliations:** Gamaleya Research Institute of Epidemiology and Microbiology, Russian Academy of Medical Sciences, 18, Gamaleya Str., 18, Moscow, Russia, 123098; Joint Institute for High Temperatures, Russian Academy of Sciences, Izhorskaya Str., 13/2, Moscow, Russia, 125412; Max Planck Institute for Extraterrestrial Physics, Scheinerstrasse, 1, Munich, Germany, 81679; Institute for Biomedical Problems, Russian Academy of Sciences, Horoshevskoe sh., 76 A, Moscow, Russia, 123007

**Keywords:** non-thermal plasma, protein p53, apoptosis

## Abstract

Non-thermal plasma (NTP) consists of a huge amount of biologically active particles, whereas its temperature is close to ambient. This combination allows one to use NTP as a perspective tool for solving different biomedical tasks, including antitumor therapy. The treatment of tumor cells with NTP caused dose-dependent effects, such as growth arrest and apoptosis. However, while the outcome of NTP treatment has been established, the molecular mechanisms of the interaction between NTP and eukaryotic cells have not been thoroughly studied thus far. In this work, the mechanisms and the type of death of human colon carcinoma HCT 116 cells upon application of non-thermal argon plasma were studied. The effect of NTP on the major stress-activated protein p53 was investigated. The results demonstrate that the viability of HCT116 cells upon plasma treatment is dependent on the functional p53 protein. NTP treatment caused an increase in the intracellular concentration of p53 and the induction of the p53-controlled regulon. The p53-dependent accumulation of active proapoptotic caspase-3 was shown in NTP-treated cells. The study was the first to demonstrate that treatment of human colon carcinoma cells with NTP results in p53-dependent apoptosis. The results obtained contribute to our understanding of the applicability of NTP in antitumor therapy.

## INTRODUCTION

Non-thermal plasma (NTP) is a flow of partially ionized gas obtained under atmospheric pressure that has a macroscopic temperature that is close of the ambient temperature [1]. The potential of using NTP for medical purposes started to be intensively investigated about 10 years ago, although the first studies in the field (predominantly in Russia) began much earlier [2–4].

A non-thermal plasma torch consists of charged particles, neutral active particles (including free radicals and metastable particles), and ultraviolet radiation. The biological effects of NTP are attributed to the synergistic action of the aforementioned factors, whereas the subthreshold concentration of each component in most cases does not alter biological objects [5, 6].

There has been considerable interest in the potential use of NTP as an antibacterial agent, since NTP has been found to possess nonspecific bactericidal activity, which enables one to use NTP to sterilize thermosensitive surfaces and sanitize tissues, including wound surfaces [7–9]. Another potential field of application for NTP is antitumor therapy. Thus, the selectivity of the cytotoxic effect of plasma on various human cell types and the opportunity of selecting particular conditions that would provide selective death of a certain type of tumor cells have been reported [10, 11]. The exposure of tumor cells to NTP has been shown to result in cell cycle delay and induction of apoptosis [12–14]. 

Opposite to the final effects caused by NTP treatment of the cells, the molecular mechanisms underlying the interaction between NTP and eukaryotic cells remain poorly studied. These data are required to elucidate the nature of the selective effect of NTP with respect to tumor cells and to determine the range of applicability of NTP. Therefore, our work aimed at studying the molecular mechanisms of the action of NTP on tumor cells and at determining the type of cell death in the cells subjected to NTP treatment.

## METHODS

**Cell lines and growth conditions**

Two sublines of human colon cancer cells (HCT116) were used in this study: HCT116(р53+/+)-ConA-lacZ subline with an active * р53* gene and the β-galactosidase reporter gene under the control of a p53-dependent promoter, and HCT116(р53-/-)-ConA-lacZ subline that had deletions of both copies of the gene encoding the p53 protein. The HCT116(р53+/+) and HCT116(р53-/-) cell lines were kindly provided by A.V. Gudkov (Roswell Park Cancer Institute, USA). The cells were grown in a DMEM medium supplemented with a 10% fetal bovine serum (Hyclone, USA), 1 mg/ml glutamine (PanEco, Russia), 50 U/ml penicillin, and 50 µg/ml streptomycin (PanEco, Russia) at 37°С in a 5% CO _2_ atmosphere. Cells were seeded at a 1 : 6 ratio on day 2 after the monolayer became confluent.

**Counting the number of live cells**

The cell survival rate was determined spectrophotometrically 25 h following the NTP treatment using the intensity of staining the live cells with a methylene blue dye. Optical density was measured at 540 and 620 nm. The relative number of cells that survived was calculated using the formula *х * = *ОЕ*_620 _ – *ОЕ*_540_ .

**Assessment of the activity of р53 under the control of a promoter based on the expression of the β-galactosidase reporter gene**

After the culture medium was removed, a lysis buffer containing a β-galactosidase substrate (1 mM MgCl _2_ , 0.25 M Tris HCl, pH 7.4, 0.02% NP40, 2 g/l *o* -nitrophenyl-β- *D* -galactopyranoside) was added to the cells. Following incubation for 30 min at 37°С, the β-galactosidase activity level was determined spectrophotometrically by measuring the optical density of the solution at 414 nm.

**NTP source**

A source of non-thermal argon microwave plasma MicroPlaSter β was used for the experiments. The NTP source contained a 2.45 GHz current generator, a burner, and a gas (argon) supply system. The device uses two regimens, the argon plasma regimen and the regimen including the flow of a nonionized argon gas. The burner is capable of generating a highly stable low-power (60–150 W) plasma flow (torch) with a low rate of gas flow (4–8 l/min). The argon plasma torch has a length of about 5 cm and a diameter of 3.5 cm. The distance of the treated surfaces from the plasma source was equal to 2±0.2 cm. At this distance, the torch temperature was 36±2 °C.

**NTP treatment of cells**

A day prior to the experiment, the cells were seeded into 3-cm-diameter culture dishes (2 × 10 ^5 ^ cells/dish). On the next day, just before the NTP treatment, the cultivation medium was removed and a 0.5-mm layer of the medium was left. The dishes were placed at a distance of 2 cm from the plasma torch and treated with NTP during the time specified below. Immediately after treatment, a fresh cultivation medium was added and the dishes were placed into a CO _2_ incubator. The number of viable cells and activity of the β-galactosidase reporter gene were determined 24 h following the treatment.

**Assessment of caspase-3 activity level**

Caspase-3 activity was measured using antibodies specific to the active protein form conjugated to the fluorescent dye fluorescein isothiocyanate (FITC, BD Pharmingen, USA). Eighteen hours following the treatment with plasma, the cells were collected and precipitated via centrifugation at 1200 rpm for 10 min. A BD Cytofix/Cytoperm™ Fixation/Permeablization Kit (BD Pharmingen) was used for cell fixation and permeabilization. Intracellular staining of the active form of caspase-3 was carried out according to the manufacturer’s (BD Pharmingen) protocol. Fluorescence was detected by flow cytofluorimetry using a BeckmanCoulter FC-500 instrument.

**Assessment of p53 activity level**

The p53 level was measured using anti-p53 antibodies conjugated to the fluorescent dye phycoerythrin (BD Pharmingen, USA). A day prior to the experiment, 2 × 10 ^5^ HCT116(р53+/+)-ConA-lacZ and HCT116(р53-/-)-ConA-lacZ cells were seeded into each of two 3-cm culture dishes. The next day, when the cells reached a 60–80% confluence, the cultivation medium was removed from the dishes and a 0.3 mm layer of the medium was left to preserve the cells from desiccation during the treatment. The cells were treated with plasma for 2 min, and a fresh DMEM medium was added immediately after treatment. After incubation for 6 h, the cells were collected and precipitated by centrifugation (10 min, 1200 rpm). A BD Cytofix/Cytoperm™ Fixation/Permeablization Kit (BD Pharmingen) was used for cell fixation and permeabilization. Intracellular staining of p53 protein-3 was carried out according to the manufacturer’s (BD Pharmingen) protocol. Fluorescence was detected by flow cytofluorimetry using a BeckmanCoulter FC-500 instrument.

**Statistics**

**Fig. 1:**
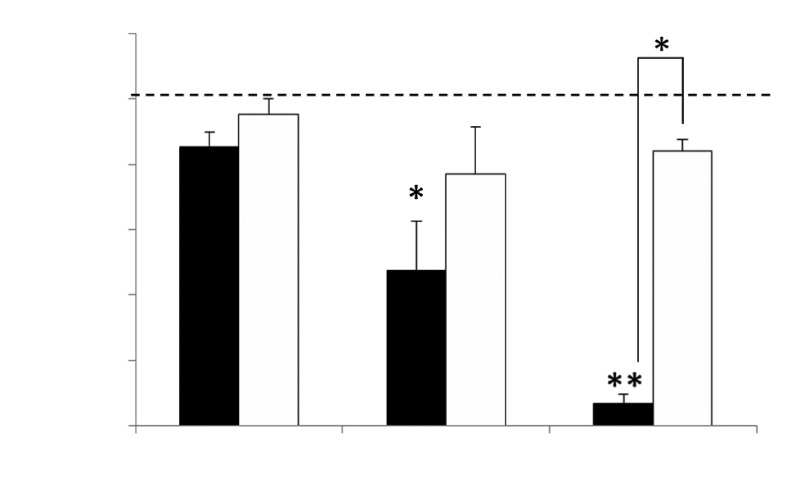
Study of survival of HCT116(p53+/+) cells after NTP (black columns) and non-ionised argon (white columns) treatment as a function of exposure time. The percentage of living cells against the intact control (dashed line) is shown. Mean values ± SD are given. * – p < 0.05, ** – p < 0.005 (as compared to the intact cells).

All experiments were performed using duplicate samples and repeated at least three times. The mean values and standard errors were calculated with the Excel software (Microsoft Office 2007).

## RESULTS AND DISCUSSION

**NTP treatment causes dose-dependent death of HCT116 cells**

The ability of NTP to activate the transcription factor p53 and induce the development of p53-dependent programs leading to apoptotic death is presumably one of the possible reasons behind tumor cell death after treatment with NTP.

The human colon cell line HCT116(р53+/+) containing the functional *р53 * gene was selected to verify this assumption. The ability of NTP to cause the death of these cells was determined at the first stage. HCT116(р53+/+) cells were treated with NTP with different exposure times. The number of cells alive was determined 24 h following the treatment. Nontreated cells and the cells treated with nonionized argon during the corresponding time period were used as controls. Treatment with NTP for 2 min resulted in no statistically significant decrease in the number of cells alive ( *Fig. 1* ). A twofold ( *p* < 0.01) and 14.5-fold ( *p* < 0.005) decrease in the number of cells alive was observed after a 5- and 7-min treatment, respectively, compared to the intact control cells. A decrease in the number of cells alive after plasma treatment for 7 min was statistically significantly different from the effect of non-onized argon ( *p* < 0.01). Treatment with nonionized argon resulted in a decrease in the number of live cells compared to that of the control cells; however, this was statistically negligible at all exposure times. The reduction in the number of cells alive can presumably be attributed to the consequences of the gas flow, which could result in desiccation of the cultivation medium, or some other nonspecific effects.

Thus, we have demonstrated that treatment with NTP leads to a decrease in the number of live cells; the intensity of the cytotoxic effect depends on the duration of treatment with the plasma flow. The treatment with non-ionized argon caused a smaller decrease that was independent of the exposure time. The results allow to conclude that the cytotoxicity of NTP is due to the specific effect of ionized NTP particles on eukaryotic cells.

**Protein p53 and p53-dependent elements are activated in НСТ116 cells treated with NTP**

Protein p53 is known to be one of the major stress-activated transcriptional regulators; its activation can initiate the development of a number of programs inducing cell death.

**Fig. 2:**
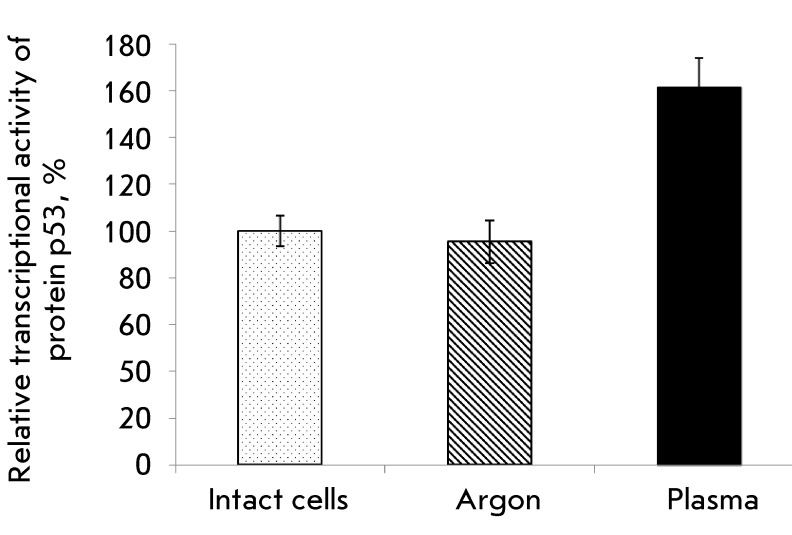
The relative transcriptional activity of protein p53 in HCT116(p53+/+) cells treated with NTP. Mean values ± SD are shown.

The effect of NTP on p53 activity was studied using HCT116 cell sublines (HCT116(р53+/+)-ConA-lacZ). The *lacZ* reporter gene encoding bacterial β-galactosidase was inserted into the genome of HCT116(р53+/+)-ConA-lacZ cells. The expression of the reporter gene was controlled by the p53-dependent promoter. The use of this reporter system allows one to determine the transcriptional activity of protein 53 based on the β-galactosidase activity level. Previously obtained data were used to determine the sub-toxic time of treatment of the cells with NTP, which does not result in pronounced cell death (2 min). The β-galactosidase gene expression level was determined spectrophotometrically 24 h following the treatment with NTP. Cells treated with a nonionized argon flow were used a controls. The 2-min treatment of cells with NTP caused a statistically significant increase in the β-galactosidase activity level, attesting to the enhancement of p53 transcriptional activity in HCT116 cells ( *Fig. 2* ).

**Fig. 3:**
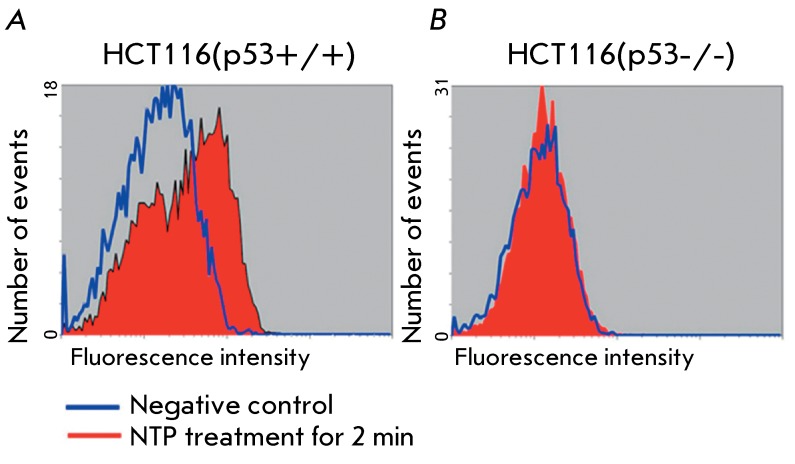
Intracellular concentration of protein p53 in HCT116(p53+/+) (А) and HCT116(p53-/-) (B) cells, intact or treated with NTP for 3 min.

The amount of p53 was additionally determined via flow cytofluorimetry using fluorescently labelled monoclonal anti-p53 antibodies. The HCT116(р53-/-) cells that had deletions of both copies of the *р53 * gene were used as the control cell line. The 2-min treatment with NTP was shown to result in a statistically significant increase in the amount of p53 in HCT116(р53+/+) cells compared to that in the intact cells ( *Fig. 3A* ). As could have been be expected, NTP treatment did not alter the nonspecific signal in HCT116(p53-/-) cells ( *Fig. 3B* ).

**Fig. 4:**
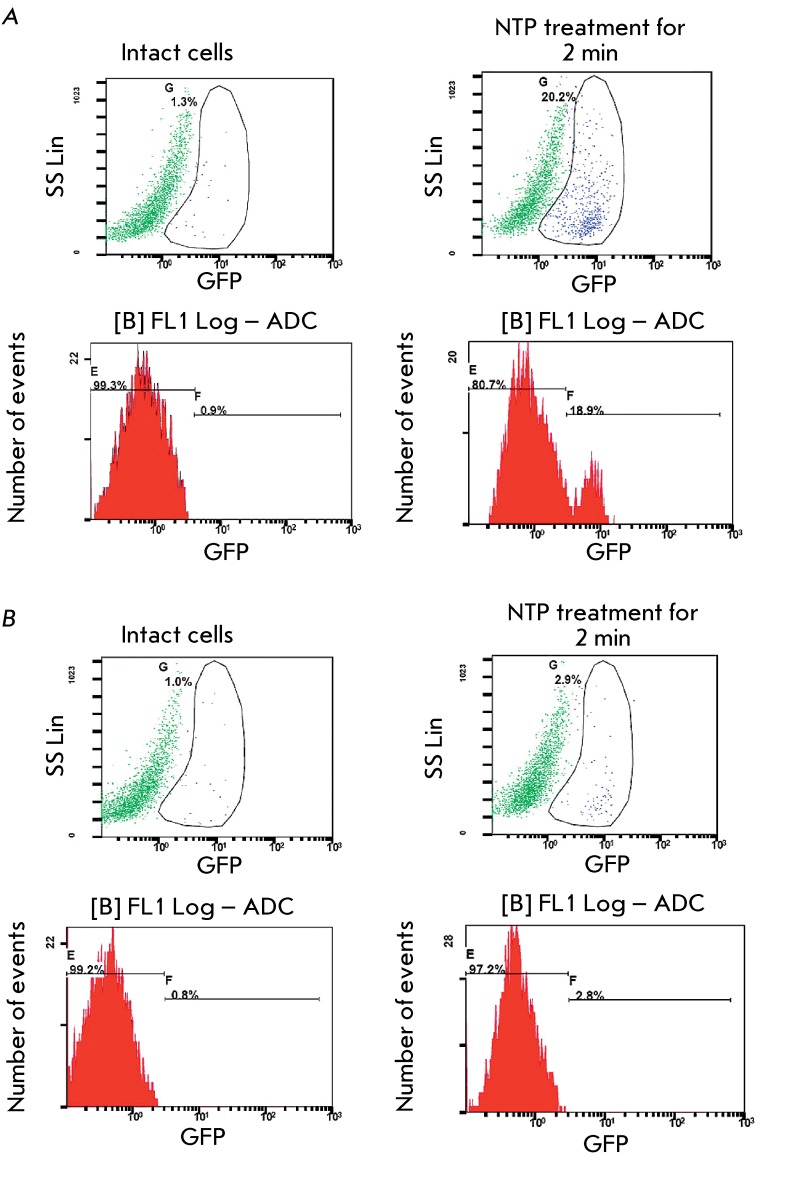
Amounts of active caspase-3 in HCT116(p53+/+) (А) and HCT116(p53-/-) (B) cells, intact or treated with NTP for 3 min.

Thus, it was ascertained that treatment of eukaryotic cells with NTP at sub-toxic exposure time results in a statistically significant ( *p * < 0.05) increase in the amount of p53 and enhancement of its transcriptional activity.

**Treatment with NTP induces apoptotic death of НСТ116 cells**

The type of cell death induced by NTP treatment had to be identified at the final stage. Apoptosis initiation through the activated p53-dependent pathway is one of the major cell death mechanisms that are known thus far [15]. Effector caspase 3 is one of the key enzymes activated upon apoptosis [16]. The activation of this protein is an integral feature of the final stages of apoptotic cell death. The level of activated caspase 3 in HCT116(p53+/+) and HCT116(p53-/-) cells treated with NTP was determined to reveal the association between p53 activation and cell death in HCT116.

A significant (up to 20%) increase in the percentage of cells containing active caspase 3 was observed in the HCT116(p53+/+) cell population treated with NTP for 2 min ( *Fig. 4A* ), whereas the NTP treatment in HCT116(p53-/-) cells had no such effect ( *Fig. 4B* ). It can be thus concluded that NTP treatment of cells results in p53-dependent activation of the main effector proapoptotic caspase 3.

Summarizing the results, one can arrive at the conclusion that NTP treatment of human cells induces activation of protein p53, the main regulator of the cellular stress response, and induces the expression of p53-dependent genes (including caspase 3), thus initiating cell death via the apoptotic pathway. Apoptosis induced by NTP was first demonstrated to occur via the p53-dependent pathway. Based on data pertaining to the enhancement of the transcription of the *p53* gene and p53-regulated *p21* gene in human hepatoma (Hep2G cells), it has been assumed that p53 participates in the cellular response to NTP treatment [17]. However, no direct evidence to support the existence of any association between the cell survival rate and presence of functional protein p53 has been obtained. Our results correspond to the induction of the β-catenin signalling pathway in human colon cancer cells treated with NTP, since this pathway is associated with the p53-dependent signalling cascade [18]. Generation of reactive oxygen species (ROS) is another signalling system participating in the cellular response to NTP treatment [14]. Intracellular ROS that interact with the components of the signalling pathways (such as protein kinases, phosphatases, and transcription factors) in a direct or mediated manner act as secondary signalling molecules, which participate in cell cycle regulation and affect the final outcome of the events induced by p53 activation [19].

However, the sequence of signalling events occurring in a cell in response to NTP treatment has not been elucidated thus far. First of all, the type of damage resulting in p53 activation has not been thoroughly ascertained. A number of studies attest to the possibility of DNA injuries as a factor inducing apoptosis in cells treated with NTP. Thus, the action of a dielectric barrier discharge as an air plasma source on MCF10A breast cancer cells results in phosphorylation of histone H2A, which is a marker of the emergence of DNA double-strand breaks [14]. However, these results are inconsistent with data that were obtained on prokaryotes and purified DNA samples and attest to the fact that the amount of double-strand breaks caused by NTP treatment is minimal [20–23]. The authors interpreted this inconsistency by assuming that DNA double-strand breaks may be caused by the NTP-induced formation of intracellular ROS [14]. Damages to the cytoplasmic membrane may be another potential signal for apoptosis development. For instance, activation of acid sphingomyelinase caused by membrane damage and an increased ceramide production may result in the development of both p53-dependent and independent apoptosis [24]. Experimental data demonstrating that it is the surface cell structures (in particular, membrane) that are the major target of active NTP particles support this mechanism of apoptosis initiation [20, 25–27]. However, no evidence in favor of this mechanism of apoptosis initiation has been obtained thus far; the details of the events occurring in the cell immediately after treatment with NTP remain to be elucidated. Meanwhile, it is obvious that for a successful application of NTP for medical purposes, a thorough understanding of what signalling events are induced by NTP depending on the dose and type of plasma radiation is required, since it is this knowledge that would allow one to optimize the treatment parameters and achieve the desired effect.

## CONCLUSIONS

It has been demonstrated that the survival rate of HCT116 colon cancer cells treated with NTP depends on the presence of the functional protein p53. NTP treatment increases the intracellular concentration of p53 and induces expression of p53-regulated genes, in particular, the major proapoptotic caspase 3. It has thus for the first time been shown that the treatment of colon cancer cells with argon NTP induces p53-dependent apoptosis. These results are of significance for better insight into the potential of using NTP as an antitumor agent. 

## Abbreviations

NTP: non-thermal plasma
